# Multifunctional Role of Poly-L-Lysine in Plasmonic Metal Nanostructures

**DOI:** 10.3390/nano16211416

**Published:** 2026-11-04

**Authors:** Anna Lizunova, Dina Kostina, Anna Shaikhtdinova, Arina Sanatulina, Ekaterina Kameneva, Olesya Vershinina, Konstantin Pilyushenko, Elizaveta Marushko, Ilya Tsaplin, Egor Khramov, Victor Ivanov

**Affiliations:** Moscow Center for Advanced Studies, 20 Kulakova Str., 123592 Moscow, Russia; dinafrolova007@gmail.com (D.K.);

**Keywords:** Poly-L-lysine, plasmonic nanostructures, localized surface plasmon resonance, dielectric spacer, photoluminescence enhancement, aluminum nanostructures

## Abstract

Poly-L-lysine (PLL) layers deposited by sequential spin coating as a multifunctional platform for plasmonic metal nanostructures were investigated. Using an identical deposition strategy, a cross-material comparison was performed for Pt, Al, and Ag nanoparticles and Ag nanowires. Increasing PLL thickness red-shifts the localized surface plasmon resonance (LSPR) by modifying the dielectric environment, and depends on nanoparticle size distribution, plasma frequency, and background dielectric contribution. Deposition of a 25 nm PLL layer results in absorption shifts of 63, 57, 28, and 45 nm for Al, Pt, and Ag nanoparticles and Ag nanowires, respectively. Mie theory simulations confirmed the experiments and revealed that larger particles exhibit stronger long-wavelength shifts. PLL-coated Al nanostructures preserved their optical response and photoluminescence-enhancing functionality after immersion in deionized water for up to 340 min. As a dielectric spacer, PLL controls the interaction between ZnO and Al nanoparticles and increased the ultraviolet (UV) photoluminescence enhancement factor of ZnO at 377 nm up to 2.1-fold relative to pure ZnO. The developed Al/PLL platform produced pronounced quenching of intrinsic IgG fluorescence at 330 nm. These findings established PLL coatings as a versatile unified strategy for precise spectral tuning of plasmonic resonances, controlling emitter/metal interactions, and stabilizing Al nanostructures for UV photonic and bioanalytical sensing.

## 1. Introduction

Plasmonic nanostructures based on metals such as aluminum (Al), silver (Ag), gold (Au), and platinum (Pt) play a central role in modern nanophotonic devices [1,2,3,4]. Their localized surface plasmon resonances, which arise from collective oscillations of conduction electrons in a small, limited volume near the nanoparticle surface, enable strong near-field enhancement and increase the excitation and emission rates of nearby emitters, leading to pronounced fluorescence amplification effects [5,6,7,8]. Furthermore, these resonances exhibit high sensitivity to the surrounding dielectric environment, making plasmonic systems particularly attractive for applications in biosensing, bioimaging, and light/matter interactions in the ultraviolet (UV) and visible spectral ranges [9,10,11,12,13]. Since the spectral characteristics of LSPRs are governed not only by nanoparticle morphology and intrinsic material properties but also by the local refractive index [14,15,16], surface functionalization represents an effective approach for tailoring plasmonic responses [17,18,19]. Recent plasmonic architectures further demonstrate how controlled coupling between localized and propagating plasmon modes can be exploited for integrated optical functionality, including on-chip polarization analysis and self-referenced refractive-index sensing [20,21].

Polymeric coatings represent a versatile strategy for modulating plasmonic behavior because they offer adjustable thickness and compatibility with metal surfaces [22]. Such coatings can improve mechanical stability, reduce aggregation, provide barrier protection, increase adhesion, and introduce functional groups for subsequent modification or bioconjugation [23,24,25]. In addition, polymer shells act as nanoscale dielectric layers that modify LSPR modes by changing the local refractive index near the nanoparticle surface [26,27,28]. Therefore, these properties make polymer coatings particularly attractive for precise control of plasmonic responses through nanoscale engineering of the local dielectric environment.

Poly-L-lysine (Figure 1) is a cationic polypeptide widely used for functionalization of metal and oxide nanostructures due to its strong surface adhesion and positive charge [29,30,31].

PLL coatings are capable of forming uniform and stable layers and exhibit strong affinity for both metal and metal/oxide surfaces [32,33]. Previous studies have demonstrated that PLL-coated metal nanoparticles show improved colloidal stability and are well suited for biomedical applications [29,34,35]. This property is particularly important for reactive plasmonic metals such as aluminum, which, despite supporting strong ultraviolet plasmon resonances, is prone to rapid oxidation and hydroxylation in aqueous environments [36,37,38,39,40]. Moreover, aluminum nanostructures can undergo rapid corrosion in water under ultraviolet illumination (e.g., at 266 nm) and without it, leading to significant degradation of plasmonic properties within tens of seconds, even at relatively low excitation powers [38]. Therefore, the development of dense hydrophilic polymer barriers is of considerable interest for improving the aqueous stability of aluminum nanostructures, and PLL is especially attractive due to its adsorption behavior and chemical functionality [22,31,41].

In addition to providing chemical protection, polymer layers such as PLL enable precise nanoscale control over the separation distance between metal nanostructures and nearby emitters [42,43,44]. This parameter is critical because plasmon-enhanced fluorescence strongly depends on the spacer thickness. For instance, systems employing polyelectrolyte multilayers have shown that the photoluminescence intensity of upconversion nanoparticles reaches a maximum at a separation distance of approximately 8 nm, with an enhancement factor of up to ~22.6, followed by a decrease at larger distances [42]. Similarly, studies of Rhodamine 6G in proximity to gold nanoparticles with poly(methyl methacrylate) (PMMA) spacer layers (1.5–21 nm) demonstrate that photoluminescence (PL) is enhanced but gradually decreases as the spacer thickness increases [43]. Furthermore, polymer-coated plasmonic nanoparticles, such as PLL-coated Ag nanoparticles with a shell thickness of ~4 nm, can significantly enhance PL by maintaining an optimal separation distance and suppressing nonradiative quenching [44]. These findings indicate that controlling the emitter/metal distance with polymer layers is an effective strategy for tuning plasmon-enhanced photoluminescence.

ZnO nanostructures are widely investigated as UV–active materials due to their high quantum efficiency, strong intrinsic emission, high redox potential, chemical and physical stability, and low cost [45,46,47,48]. However, pure ZnO often suffers from rapid electron/hole recombination and limited light absorption efficiency [49]. Incorporation of plasmonic metal nanoparticles can significantly enhance its optical response through plasmon-exciton coupling at the interface [49,50,51]. For example, ZnO nanorods decorated with Cu or Al nanoparticles exhibit enhanced near-band-edge emission, with increases of approximately 7.6× and 2×, respectively [52]. Similarly, Ag/ZnO and Au/ZnO systems demonstrate strong enhancement of near-band-edge UV emission by approximately 60× and 17×, respectively [50]. In addition, Au/ZnO nanocomposites show substantially improved device performance, including up to ~80× enhancement in UV photodetector response [51]. These results confirm that ZnO/metal structures effectively enhance UV emission and related optoelectronic properties. Therefore, the introduction of a thin polymer interlayer between ZnO and metal nanostructures provides an additional means to control and stabilize these interactions.

Polymer coatings also enable controlled tuning of LSPR characteristics by modifying the local dielectric environment and adjusting the separation between metal nanoparticles and surrounding materials [43,53]. For example, Au nanoparticles separated from Rhodamine 6G by PMMA spacer layers exhibit a red shift of the plasmon resonance peak from 530 nm to 580 nm as the spacer thickness increases from 1.5 to 21 nm [43]. However, systematic experimental studies on LSPR tuning with polymer coatings across different plasmonic nanostructures remain limited. This lack of data restricts understanding of how polymer thickness and composition influence plasmonic behavior in metal/semiconductor systems.

The aim of this work is to systematically investigate the influence of Poly-L-lysine layer thickness on the optical properties of plasmonic metal nanostructures. Specifically, we examine the effect of sequential PLL deposition on the positions of absorption resonances in aluminum, platinum, and silver nanostructures across the ultraviolet and visible spectral ranges. In addition, we investigate PLL as a dielectric spacer of controlled thickness in ZnO/PLL/Al systems to enhance photoluminescence and assess its ability to protect aluminum nanostructures in water. We further evaluate the effect of Al/PLL nanostructures on the intrinsic PL of immunoglobulin G (IgG).

## 2. Materials and Methods

### 2.1. Materials

Poly-L-lysine (PLL, 0.1 wt.% in H_2_O, Sigma-Aldrich, St. Louis, MO, USA) was used as a film-forming polymer. For spin-coating deposition, the PLL solution was diluted with 2-propanol (Hipersolv Chromanorm, VWR International GmbH, Darmstadt, Germany) in a volume ratio of 1:3 (*v*/*v*).

Colloidal aluminum nanoparticles (Al NPs) with a concentration of 0.18 g/L and an average particle size of 55 ± 25 nm were synthesized by the electrical wire explosion method and dispersed in chromatographically pure isopropyl alcohol (ISO) containing citric acid (0.05 g/L) as a stabilizing agent, following the procedure described in [54].

Zinc oxide nanoparticles (ZnO NPs) with an average particle size of 27 ± 7 nm were prepared from a commercial ZnO dispersion (Sigma-Aldrich, Buchs, Switzerland; 40 wt.%) according to the protocol reported in [54], resulting in a final colloidal concentration of 1.1 g/L.

Platinum nanoparticles were produced using a pulsed-periodic gas discharge method [55] by electric erosion of cylindrical Pt electrodes. The electrodes, made of highly pure material (99.9999%), had outer and inner diameters of 8 mm and 7 mm, respectively. The synthesis was carried out in an argon environment with a chemical purity level of 6.0 (Ar 6.0), under an overpressure of 0.6 atm. A voltage of 1.5 kV was applied to the electrodes; the pulse repetition rate was 500 Hz, and the energy stored in the 40 nF capacitor was discharged through the interelectrode gap. The synthesis was carried out at a gas flow (Ar 6.0) of 50 mL/min. Primary nanoparticles generated within the interelectrode gap were carried by the argon stream through the gas path and then subjected to in-flow thermal treatment in a tube furnace heated to 985 °C. The sintered nanoparticles were subsequently focused through a nozzle and deposited on a quartz glass substrate using dry aerosol printing [56]. The formation of the Pt film took place at a constant distance from the nozzle to the substrate of 2.5 mm and a fixed interline spacing (step between lines) of 150 µm. Film absorption was controlled through coordinated variation of the substrate speed at 6000 µm/s.

Silver nanoparticles (Ag NPs) were synthesized by chemical reduction using sodium borohydride as a reducing agent and polyvinylpyrrolidone (PVP) as a stabilizer. PVP (Mw = 10 kDa) was dissolved in 100 mL of deionized water under magnetic stirring until complete dissolution, followed by the addition of 1 g of silver nitrate. The mixture was stirred until a homogeneous solution was obtained. Separately, 0.2 g of sodium borohydride (NaBH_4_) was dissolved in 10 mL of cold deionized water (5 °C), yielding a 0.529 M solution. The freshly prepared NaBH_4_ solution (8 mL) was rapidly injected into the AgNO_3_/PVP solution under vigorous stirring, resulting in an intense reduction process and the formation of silver nanoparticles. Stirring was continued for 10 min. Purification of Ag NPs was carried out by centrifugation of six 20 mL portions of the as-prepared dispersion at 15,000 rpm for 60 min. After removal of the supernatant, the nanoparticles were washed twice by sequential precipitation and redispersion in isopropyl alcohol to remove residual PVP. Finally, the purified Ag NPs were redispersed in ISO.

Silver nanowires (Ag NWs) were synthesized using a modified polyol method in a spatially confined reaction environment. Polyvinylpyrrolidone (PVP, Mw = 360 kDa, Sigma-Aldrich, St. Louis, MO, USA) was used as a polymer stabilizer. Silver nitrate (AgNO_3_) and iron(III) chloride hexahydrate (FeCl_3_·6H_2_O) were purchased from Lenreaktiv (St. Petersburg, Russia). Ethylene glycol, isopropyl alcohol, and acetone were obtained from Chimmed (Moscow, Russia). All chemicals were used as received without further purification. In a typical synthesis, 0.090 g of PVP was dissolved in 11 mL of ethylene glycol, and 0.045 g of AgNO_3_ was dissolved separately in 1.5 mL of ethylene glycol. After complete dissolution, 0.625 mL of a 3 mmol/L FeCl_3_ solution was added to the PVP solution. The resulting mixture was heated to 170 °C in a glass reactor placed in an oil bath under constant stirring. After achieving a homogeneous solution, the AgNO_3_ solution was injected, initiating the reduction of silver ions. The reaction temperature was maintained for 90 min, resulting in the formation of silver nanowires with a high aspect ratio. The obtained suspension was mixed with acetone in a 3:1 volume ratio and centrifuged at 1000 rpm for 3 min to remove silver nanoparticles. The precipitate was redispersed in 13 mL of isopropyl alcohol and further purified by three sequential washing steps via centrifugation at 2000 rpm for 5 min. A final centrifugation at 500 rpm for 5 min was performed to sediment Ag NW aggregates, which were subsequently removed.

### 2.2. Fabrication of Nanostructured Films

All nanostructured films were fabricated on quartz substrates (1.2 × 4 cm^2^) using a laboratory spin coater (EZ4, Schwan Technology). For each deposition step, 15 µL of the corresponding suspension was applied at 1000 rpm for 30 s with an acceleration of 500 rpm/s. Multilayer nanosystems were fabricated by sequential spin-coating with intermediate drying steps. Al NPs, Ag NPs, Ag NWs, and ZnO films were deposited using 15, 30, 8, and 3 sequential layers, respectively, resulting in absorption of 0.10 at 225 nm (Al), 0.05 at 413 nm (Ag NPs), 0.05 at 372 nm (Ag NWs), and 0.03 at 377 nm (ZnO). Platinum films exhibited an absorption (abs) of 0.17 at 258 nm. All deposition procedures were carried out at room temperature under ambient conditions.

### 2.3. Deposition of Poly-L-Lysine Layers

PLL films with systematically varied numbers of sequentially deposited layers were prepared on quartz substrates or pre-formed metal nanostructures. For the main comparative experiments, coatings consisting of 1, 5, or 10 PLL layers were used. For Al and Pt nanoparticle films, additional intermediate layer numbers (2, 3, 7) and an extended series were investigated to analyze the thickness-dependent plasmonic response. For each deposition step, 15 µL of the corresponding suspension was applied at 1000 rpm for 30 s with an acceleration of 500 rpm/s. After each PLL deposition step, the samples were briefly dried under ambient conditions before deposition of the subsequent layer. The number of deposited layers was systematically varied to control the effective polymer thickness and to investigate its influence on the optical properties of the nanostructured films. The thickness of PLL films was determined by spectroscopic ellipsometry: 5 sequential layers correspond to an effective thickness of 17 nm, whereas 10 layers form a polymer film with a thickness of 25 nm. The corresponding thicknesses of prepared PLL coatings with 1, 2, 3, and 7 layers are presented in Table S1 in the Supplementary Information. To study the role of PLL in plasmon-enhanced photoluminescence, several types of structures were fabricated on quartz substrates, including single-component films (PLL, Al, and ZnO), bilayer systems (PLL/Al, ZnO/PLL, and ZnO/Al), and three-layer systems (ZnO/PLL/Al). In the bilayer ZnO/Al structures, Al nanoparticle layers were first deposited onto quartz substrates, followed by ZnO nanoparticle layers. For ZnO/PLL/Al structures, Al nanoparticle layers were first deposited onto quartz substrates, then PLL layers were deposited onto the Al film, and finally ZnO nanoparticle layers were deposited onto the PLL-coated Al film. The systematic variation in the number of PLL layers enabled evaluation of the influence of polymer thickness on the optical response. A schematic illustration of the multilayer PLL deposition onto metal nanostructures is shown in Figure 1.

### 2.4. IgG Sample Preparation

To prepare a homogeneous IgG film, the PLL/Al sample was incubated for 2 h in a phosphate–citrate buffer at pH 4.4 containing human immunoglobulin G (IgG) at a concentration of 0.5 mg/mL, then subjected to denaturation in a vertical position at 80 °C for 15 min.

### 2.5. Water Stability Test

The stability of PLL-coated aluminum nanostructures in aqueous environments was evaluated by immersing the samples in 20 mL of deionized water at room temperature (22 ± 2 °C) for different time intervals (10, 40, 100, and 340 min). Ultraviolet–visible (UV–vis) absorption spectra were recorded after each immersion step. After exposure, the samples were removed and dried in an oven (BINDER GmbH, Tuttlingen, Germany) at 80 °C. UV–vis absorption spectra of Al and PLL/Al films after water exposure were compared with reference samples that were not subjected to aqueous treatment. To further assess the protective role of PLL, photoluminescence measurements were performed on ZnO-coated PLL/Al structures before and after water exposure.

### 2.6. Optical and Structural Characterization

The size, morphology, and crystal structure of the primary nanoparticles were characterized by transmission electron microscopy (TEM) using a JEOL JEM-2100 microscope (JEOL Ltd., Akishima, Tokyo, Japan) operated at 200 kV. Surface morphology of the deposited nanostructured films was analyzed by scanning electron microscopy (SEM) using a JEOL JSM-7001F microscope (JEOL Ltd., Akishima, Tokyo, Japan). UV–vis absorption spectra were recorded using a JASCO V-770 spectrophotometer (JASCO Corporation, Hachioji, Tokyo, Japan) in the 200–700 nm wavelength range. For each experimental condition, three independently prepared replicate samples were fabricated and measured to assess the repeatability of the optical response. Measurements were performed in continuous scanning mode at a scan speed of 40 nm/min, with a spectral bandwidth of 1.0 nm, a response time of 0.06 s, and a data interval of 0.1 nm, with further averaging over 10 spectra. To isolate the plasmonic contribution, the spectra of PLL-coated metal structures were corrected by subtracting the corresponding spectra of PLL layers deposited on bare quartz substrates under identical conditions. The UV–vis absorption spectra of the corresponding reference PLL films consisting of 1, 5, and 10 sequentially deposited layers are presented in Figure S1 of the Supplementary Materials. The corrected spectra were subsequently normalized to their maximum absorbance for comparison. Photoluminescence measurements were performed using a JASCO FP-8300 spectrofluorometer (JASCO Corporation, Hachioji, Tokyo, Japan) with an excitation wavelength of 325 nm and excitation and emission bandwidths of 10 and 10 nm. The thickness of PLL films deposited by sequential spin-coating was measured using a SER 800 spectroscopic ellipsometer (SENTECH Instruments GmbH, Berlin, Germany) in the spectral range of 240–1000 nm. Experimental data processing and graphical representation were performed using Origin 2021 (OriginLab Corporation, Northampton, MA, USA). 

## 3. Results and Discussion

### 3.1. Morphology and Structural Characterization

SEM and TEM analyses were completed to evaluate the morphology, size distribution, and structural uniformity of the fabricated plasmonic and ZnO-based nanostructures and are presented in Figure 2 and Figure 3, respectively. The particle size distributions are presented in the Supplementary Materials (Figure S2).

The SEM images reveal homogeneous and continuous coverage of quartz substrates by Al, ZnO, Pt, Ag nanoparticles, and Ag nanowires without noticeable aggregation or large-scale defects, confirming the reproducibility of the deposition procedure. Such uniformity is important for reliable optical measurements because structural inhomogeneities and aggregation strongly affect the plasmonic response of nanostructured films. TEM analysis shows that Al NPs are predominantly spherical and exhibit a core/shell structure consisting of a metallic Al core and a native Al_2_O_3_ shell of approximately 3 nm thickness, with a mean particle diameter (including the oxide shell) of 55 ± 25 nm [57]. ZnO NPs are approximately spherical with a mean size of 27 ± 7 nm. Pt nanoparticle films consist of quasi-spherical particles with an average size of 105 ± 33 nm (Figure S2). Ag NPs are approximately spherical with a mean particle size of 36 ± 13 nm. Ag NWs exhibit an average diameter of 84 ± 21 nm and a length of approximately 15 µm.

### 3.2. Modulation of Plasmon Resonances via PLL Surface Coatings

PLL layers act as dielectric spacers that modify the local refractive index near the metal surface and shift the LSPR toward longer wavelengths, as schematically illustrated in Figure 1. UV–vis absorption spectra demonstrate pronounced red shifts of the LSPR peaks in all investigated systems after PLL deposition (Figure 4). For clarity, the presented spectra correspond to background-corrected absorbance (PLL on metal nanostructure subtracted with pure PLL reference with the corresponding number of layers) and are normalized to their maximum absorption. Labels “n L PLL on Pt” denote the absorbance of metal nanostructures coated with n PLL layers after subtraction of the corresponding PLL reference spectrum.

Figure 4a shows a monotonic red shift for platinum films from 258 nm for the uncoated sample to 274 nm, 284 nm, and 315 nm after deposition of 3, 5, and 10 PLL layers, respectively. This behavior demonstrates a strong dependence of the Pt optical response on the dielectric environment at the initial stages of coating growth. The dependence of the LSPR wavelength shift of Pt nanoparticles on PLL thickness is presented in Figure S5 of the Supplementary Materials. 

For aluminum nanoparticle films, the bare Al sample exhibits a single LSPR peak at 225 nm and a long wavelength tail due to a broad particle size distribution [58]. The plasmon resonance peak undergoes a red shift as the number of polymer layers increases from 1 to 10, which corresponds to an increase in dielectric layer thickness from 8 to 25 nm, correspondingly. However, this shift is non-uniform because of a saturation effect, wherein the incremental contribution of further film thickening to the plasmon resonance shift diminishes progressively with increasing thickness. After deposition of 1 PLL layer, the spectrum evolves into one maximum at 268 nm. With a further increase in PLL thickness to 10 layers, the peak shifts toward longer wavelengths, reaching 288 nm. That is, the plasmon resonance shift becomes progressively less sensitive to further increases in coating thickness once the dielectric layer extends beyond the spatial range of the plasmonic near-field. The dependence of the plasmon shift on polymer thickness, together with its exponential approximation, is presented in the Supplementary Materials (Figure S4). Silver nanostructures exhibit the same overall tendency, although the magnitude of the shift depends on the particle-size distribution. In Ag nanoparticle films, the LSPR maximum shifts from 413 nm to 436 nm and 441 nm after deposition of 5 and 10 PLL layers, respectively. In Ag nanowire films, the corresponding shift is larger, from 372 nm to 404 nm and 417 nm. The larger spectral shift observed for Ag nanowires is attributed primarily to differences in the characteristic size and particle-size distribution: Ag NWs have a larger characteristic diameter and a greater contribution of larger structures to the ensemble response compared with Ag NPs. The experimental values of the resonance peak shift are summarized in Table 1. 

In the present analysis, the Ag NW response is assigned to the transverse plasmon mode, which is primarily determined by the nanowire diameter. Longitudinal modes associated with the full nanowire length were not considered, since they are expected to occur at wavelengths on the order of micrometers. Notably, increasing the PLL thickness from 5 layers (~17 nm) to 10 layers (~25 nm) produces only a relatively small additional red shift for both Ag nanoparticles and Ag nanowires. This behavior suggests the onset of saturation of the LSPR response with increasing dielectric thickness. Similar trends have been reported for Ag nanoparticles coated with organic multilayers formed by layer-by-layer assembly of alkanethiol molecules, where the LSPR shift increased nonlinearly with layer thickness and gradually saturated at total overlayer thicknesses of approximately 20–30 nm [60]. Beyond this range, only minor additional spectral shifts were observed, indicating that the plasmonic response is mainly governed by the dielectric environment close to the nanoparticle surface [60]. A comparable tendency for the resonance shift to approach a constant value was also reported for Au nanoparticle systems coupled to graphene: the resonance shift increased as the spacer thickness grew from 0 to approximately 15 nm but showed only minor additional changes when the spacer thickness exceeded ~20 nm, consistent with the decay of electromagnetic coupling with distance [61]. A more detailed comparison with reported polymer-induced plasmon shifts is provided in Section 3.5. Therefore, the weak additional red shift at higher PLL thickness is consistent with the finite spatial extent of the plasmonic near field and with the reduced sensitivity of LSPR to dielectric changes farther from the metal surface for each of the investigated materials.

### 3.3. Numerical Simulations of the Extinction Spectra: Size, Medium, and Material Effects

In order to interpret the measured optical behavior with respect to the nanoparticle size distribution and the surrounding dielectric film thickness, the extinction spectra were simulated computationally. Numerical calculations were performed within the Mie-theory framework while accounting for the experimentally determined particle-size distributions. Extinction spectra of the Ag, Pt, and Al nanoparticle ensembles were simulated using the TEM-derived size distributions and a spherical-particle approximation (Figure 5). A uniform 3 nm-thick native alumina shell was included around each Al core. Because the Ag nanowires have a high aspect ratio and micrometer-scale lengths, the present analysis considered only the transverse plasmon mode associated with electron oscillations across the wire diameter. To retain the same Mie-theory framework, the Ag NWs were approximated by spheres with a size distribution corresponding to the experimentally determined wire-diameter distribution. This approximation captures the diameter-dependent transverse response but does not reproduce the longitudinal modes or the full anisotropic response of a nanowire. The particle-size distributions are provided in the Supplementary Materials. Accordingly, the Ag NW calculation should be interpreted as a qualitative estimate of the transverse-mode response: polarization-dependent anisotropy, longitudinal resonances, and nanowire end effects are outside the scope of the spherical approximation.

The Mie-theory analysis was performed for two limiting dielectric environments: air (n_m_ = 1.00) and a homogeneous PLL environment described by the Cauchy fit to the ellipsometric data (n_m_ ≈ 1.45 in the relevant spectral range). The PLL dispersion was represented as n(λ) = A + B/λ^2^ + C/λ^4^ and extrapolated into the ultraviolet range; the corresponding optical constants are presented in the Supplementary Materials (Figure S3). These limiting cases were used to estimate the maximum resonance displacement associated with replacing the air surrounding the nanostructures with PLL.

For the polydisperse Al nanoparticle ensemble in air, the extinction profile reveals a main peak at 194 nm, with a secondary, weak broad shoulder discernible at ~250 nm along the shallow long-wavelength tail (Figure 5a). After the Al nanoparticles were subjected to the PLL matrix, these maxima shifted to 214 and 287 nm, respectively. Thus, the calculated red shifts were 20 nm for the short-wavelength maximum and 37 nm for the long-wavelength one. For the Al ensemble, the lower-energy, longer-wavelength contribution therefore demonstrated moderate sensitivity to the surrounding dielectric medium.

To clarify the role of particle size in modulating the plasmonic response to environmental changes, additional calculations were performed for monodisperse Al nanoparticles with diameters of 30 and 100 nm, including the 3 nm native oxide shell (Figure 5a, grey and cyan curves). For the 100 nm particles, several higher-order plasmonic modes were observed because retardation and multipolar effects become significant as the particle size increases. The high-energy mode shifted from 206 to 235 nm, corresponding to a shift of 29 nm (~14.1%), whereas the low-energy peak moved from 301 to 352 nm, giving a shift of 51 nm (~16.9%). By contrast, the 30 nm particles exhibited a single dominant resonance with a less pronounced shift from 195 to 210 nm. These findings confirmed that the nanoparticle size affects the sensitivity of extinction to environmental changes.

The Pt nanoparticle ensemble exhibited a multimodal extinction spectrum (Figure 5b) with three peaks in air medium located at 254, 298, and 385 nm. A minimal shift of 8 nm for the first UV peak was observed, whereas the longer-wavelength maximum moved more noticeably from 385 to 412 nm (Δλ = 27 nm). Thus, different spectral components of the same polydisperse ensemble show markedly different dielectric sensitivities.

For Ag nanoparticles, the extinction maximum shifted from 360 nm in air to 380 nm in PLL (Figure 5c), corresponding to Δλ = 20 nm (~5.6%), whereas for Ag nanowires, the broad transverse extinction maximum shifted from approximately 385 nm in air to 425 nm in PLL, giving Δλ ≈ 40 nm. The larger shift of the Ag NW response compared with Ag NPs is consistent with the combined influence of their larger particle-size distribution, which contains a greater fraction of large structures that can support higher-order plasmon modes [62,63].

To extend the extinction analysis to the local optical response, the electromagnetic-field enhancement was calculated for a representative Al/Al_2_O_3_ core–shell nanoparticle with a total diameter of 55 nm, where the diameter includes the native oxide shell. A concentric core/double-shell model was used, consisting of a metallic Al core, a fixed 3 nm Al_2_O_3_ inner shell, and an outer PLL shell whose thickness was varied from 0 to 40 nm. Figure 5d shows a representative spatial field map calculated at an excitation wavelength of 250 nm for a 40 nm PLL shell, demonstrating localization of the enhanced field in the vicinity of the particle. Figure 5e summarizes the wavelength dependence of the field-enhancement factor for selected PLL thicknesses.

The calculations reveal a pronounced thickness-dependent distribution of the enhancement spectrum. For the uncoated Al/Al_2_O_3_ particle, the maximum field-enhancement factor reaches approximately 3.3 near 175 nm. Upon addition of PLL, the main enhancement maximum shifts toward longer wavelengths as the polymer shell becomes thicker. For a PLL thickness of approximately 30 nm, the enhancement peaks near 250 nm at about 2.8. Within the 225–325 nm spectral interval, appropriate PLL thicknesses provide local-field enhancement values up to approximately 10–15% above those of the uncoated particle. The two-parameter map in Figure 5f further visualizes this behavior and shows an extended region with enhancement factors of approximately 2.0–2.8 for excitation wavelengths of 250–300 nm and PLL thicknesses of roughly 18–38 nm.

These calculations are directly relevant to the ZnO excitation conditions used experimentally. At an excitation wavelength of 325 nm and a PLL thickness of approximately 25 nm, corresponding to 10 deposited PLL layers, the simulated local-field enhancement is of the order of 2.0. This value is consistent in magnitude with the experimentally observed ZnO photoluminescence enhancement factor of 2.1 for the ZnO/PLL(10 layers)/Al structure (Figure 6c), whereas direct ZnO deposition on Al without PLL gives an enhancement factor of 1.4. The comparison suggests that local excitation-field amplification provides an important contribution to the observed PL enhancement. The present field calculations account only for the local electric field intensity enhancement at the incident excitation wavelength and do not consider contributions of the radiative or nonradiative decay rate to emission amplification.

We consider the influence of the refractive index on the position of the plasmon-resonance absorption peak. For a small spherical nanoparticle in the quasistatic dipole approximation, the localized surface plasmon resonance condition can be approximated as

(1) Re[ εNPωres]=−2εm=−2nm2,

where *ε*_NP_ is the dielectric function of the nanoparticle, *ε*_m_ is the dielectric permittivity of the surrounding medium, and *n*_m_ is its refractive index.

Within the Drude model, the dielectric function of the metal is expressed as

(2) $$\varepsilon_{\mathrm{NP}}\left(\omega \right)=\varepsilon_{\infty }-\frac{\omega_{p}^{2}}{\omega^{2}+\mathrm{i\gamma \omega}},$$

where *ε*_∞_ represents the background contribution of bound electrons and interband transitions, *ω*_p_ is the plasma frequency, and *γ* is the damping constant. Because interband transitions contribute substantially in the visible and ultraviolet spectral ranges, particularly for Pt and Ag, *ε*_∞_ cannot generally be assumed to be equal to unity. The real part of the dielectric function is

(3) Re εNPω=ε∞−ωp2ω2+γ2.

Substitution into the resonance condition gives

(4) $$\varepsilon_{\infty }-\frac{\omega_{p}^{2}}{\omega_{\mathrm{res}}^{2}+\gamma^{2}}=-2n_{m}^{2}.$$

Therefore,

(5) $$\omega_{\mathrm{res}}=\sqrt{\frac{\omega_{p}^{2}}{\varepsilon_{\infty }+2n_{m}^{2}}-\gamma^{2}}.$$

When damping is neglected, *γ* ≪ *ω*_res_,

(6) $$\omega_{\mathrm{res}}\approx \frac{\omega_{p}}{\sqrt{\varepsilon_{\infty }+2n_{m}^{2}}}.$$

The corresponding resonance wavelength is

(7) $$\lambda_{\mathrm{res}}\approx \frac{2\mathrm{\pi c}}{\omega_{p}}\sqrt{\varepsilon_{\infty }+2n_{m}^{2}}.$$

For a finite change in the refractive index of the surrounding medium Δ*n*_m_, and writing the plasma frequency in terms of the plasma energy *E_p_* = ℏω_p_, Equation (7) gives

(8) $$\Delta \lambda_{\mathrm{res}}\approx \frac{\;n_{m}}{E_{p}\sqrt{\varepsilon_{\infty }+2n_{m}^{2}}}\Delta n_{m}$$

Equation (8) isolates the material dependence of the simplified Drude estimate, where *E_p_* is the plasma energy of the metal, *ε*_∞_ is the background dielectric contribution, *n*_m_ is the refractive index of the first dielectric environment, and Δ*n*_m_ describes the variation of the ambient refractive index. Using *n*_m_ = 1.00 for air and Δ*n*_m_ = 0.45 for the refractive-index change from air to PLL, together with the simple-Drude assumption *ε*_∞_ = 1 for all three metals; Equation (8) provides a simplified material-dependent estimate of the LSPR sensitivity. With plasma energies *E_p_* = 14.98, 9.59, and 9.01 eV for Al, Pt, and Ag, respectively, the corresponding proportionality coefficient predicts the sensitivity sequence Al < Pt < Ag [59]. However, the Mie simulations show that the magnitude of the dielectric-induced shift can not be explained solely by the metal-dependent parameters. For the Al ensemble, the main peak shifts by 20 nm. The three Pt peaks considered in Figure 5b shift by 8, 10, and 27 nm, whereas the Ag NP and Ag NW maxima shift by 20 and 40 nm, respectively (Table 1). Here, the assumption *ε*_∞_ = 1 is introduced only for the illustrative comparison of the proportionality coefficients within the simple-Drude approximation and is not used in the numerical Mie calculations. The Mie simulations instead use wavelength-dependent complex optical constants, thereby incorporating the material-specific spectral dispersion and interband contributions contained in the corresponding optical-constant datasets.

The experimental results further demonstrate that the dielectric sensitivity cannot be determined only by the plasma energy. After deposition of 10 PLL layers, the Pt maximum shifts by 57 nm, the Ag NP maximum by 28 nm, and the Ag NW maximum by 45 nm. For Al, the peak at 225 nm shifts to 288 nm after deposition of a 25 nm-thick polymer coating. For all nanoparticle coatings examined, the experimentally observed shift exceeds that predicted by the Mie model. We attribute this discrepancy primarily to interparticle interactions on the substrate, where the particles can no longer be regarded as isolated. This interpretation is corroborated by microscopic images of the nanostructured surfaces (Figure 2), which reveal that 2–10 particles lie in immediate proximity to or in direct contact with one another, so that collective plasmonic coupling necessarily comes into play. As the experimental shift is greater than the theoretical one, we therefore consider the plasmon-coupling effect to be the dominant origin of the significant experimental shift. According to Table 1, aluminum and platinum nanostructures display similar sensitivities to variations in the surrounding dielectric environment, and both exhibit a stronger dependence than silver. This behavior is attributable to the dispersion of the polymer refractive index, which rises substantially toward the ultraviolet, where the plasmon resonances of both aluminum and platinum nanoparticles are located. The Drude model predicts that the sensitivity of the plasmon peak to changes in the local environment should increase in the order Al < Pt < Ag. However, the measured plasmon peak shift increases in the order Ag < Pt < Al, indicating that the sensitivity to the thickness of the PLL coating of the plasmon resonance follows a sequence opposite to that anticipated by the model. This inversion indicates that the simplified Drude description (ε∞ = 1) does not capture the environmental response of these metals, especially in the UV region.

A major reason for the differences between the simplified Drude-based description and the experimentally measured or Mie-calculated spectral shifts is the limited applicability of the free-electron Drude approximation in the ultraviolet and visible ranges. Interband transitions contribute substantially to the complex dielectric response and cannot, in general, be represented by a universal *ε*_∞_ = 1. Lorentz-Drude and Brendel-Bormann descriptions explicitly include both free-electron and interband contributions over a broad energy range [59]. The effective high-frequency contribution is therefore model- and spectral-range dependent. For example, a Drude-Lorentz parametrization for Ag uses *ε*_∞_ = 3.7 [64]; moreover, in other studies, the same physical magnitude in the visible spectral range reveals 0.007 for silver and 0.13 for aluminum [65]. At the same time, recent spectroscopic ellipsometry and first-principles analysis of Pt demonstrate a broad interband contribution and document substantial discrepancies among previously reported Pt dielectric functions [66]. Thus, the simplified analytical estimate and the numerical Mie calculations should be regarded as distinct levels of description.

A further source of uncertainty is the choice of wavelength-dependent complex optical constants used in the simulations of extinction spectra. Published datasets are compiled from different experiments and parametrizations; the Refractiveindex.info database provides a traceable compilation of such optical-constant data [67]. Because Mie spectra depend directly on the real and imaginary parts of the dielectric constant, differences between datasets can affect the calculated peak positions, especially in spectral regions with strong interband structure.

Particle size and size distribution also provide an additional contribution. The monodisperse Al calculations directly demonstrate this effect: the 30 nm particles show a single 15 nm shift, whereas the 100 nm particles support higher-order modes with shifts of 29 and 51 nm. The Ag results are consistent with the same general tendency: Ag NWs, with a larger characteristic diameter, exhibit a larger shift than the smaller Ag NPs in both simulation and experiment. In a polydisperse ensemble, the size distribution therefore changes the relative contribution of dipolar and higher-order modes and can alter the apparent shift of the ensemble maximum. The theoretical, numerical, and experimental dielectric-induced spectral shifts are summarized in Table 1. Overall, the magnitude of the dielectric-induced plasmonic shift is governed jointly by the material-specific dielectric response (including the plasma energy and interband contributions), particle size and particle-size distribution, and the plasmon-coupling effect on the surface.

### 3.4. Applications and Water Stability of PLL-Coated Aluminum Nanostructures

Beyond dielectric tuning of the plasmon resonance, PLL coatings provide several additional functions in Al-based nanostructures. In particular, PLL acts as a protective barrier that improves the stability of aluminum nanostructures in aqueous environments, a dielectric spacer that controls the emitter–metal separation and thereby regulates the photoluminescence of ZnO, and an interfacial layer enabling modulation of the intrinsic fluorescence of biomolecules such as IgG. These functions are considered separately below. First, the protective effect of PLL on aluminum nanostructures in aqueous environments was investigated. Figure 6d compares the optical response of bare and PLL-coated Al nanoparticle films upon exposure to deionized water.

Uncoated Al films undergo rapid degradation, accompanied by the disappearance of the characteristic absorption peak near 225 nm as a result of oxidation and hydroxylation. In contrast, Al films coated with 10 PLL layers retain their characteristic plasmonic response after immersion in deionized water. Figure 6d includes the spectra of PLL-coated Al films after 40 and 340 min of immersion in addition to the initial spectrum. The spectral profiles recorded after prolonged exposure remain close to those of the initial PLL-coated Al sample, with no pronounced changes in the position of the main spectral features. In contrast, the uncoated Al film exhibits a substantial change in its optical response—the plasmon peak disappears after immersion in water. These results confirm that the PLL coating acts as an effective protective barrier, limiting direct interaction of aluminum with the aqueous environment and preserving the characteristic optical response of the Al nanostructures for at least 340 min.

After establishing the protective effect of PLL, the Al/PLL nanostructures were evaluated as substrates for enhancement of the ultraviolet photoluminescence of ZnO (Figure 6a,c). Pure ZnO exhibits an emission maximum at approximately 377 nm and was used as the reference with an enhancement factor of 1. Direct deposition of ZnO on Al nanoparticles results in only moderate PL enhancement, with an enhancement factor of 1.4. The introduction of a PLL spacer substantially increases the ZnO emission intensity: the enhancement factor reaches 1.9 for 5 PLL layers and 2.1 for 10 PLL layers, while the position of the ZnO emission maximum remains essentially unchanged. In other words, application of a 25 nm PLL layer as a dielectric spacer between metal Al nanoparticles and ZnO emitter crystals leads to a 50% improvement in photoluminescence intensity relative to a ZnO/Al sample without the interlayer.

The increase in PL intensity with PLL thickness reflects the distance-dependent nature of the interaction between the ZnO emitters and the Al nanostructures. At short emitter–metal separations, nonradiative energy transfer to the metal can contribute to fluorescence quenching, whereas increasing the spacer thickness reduces these losses while maintaining plasmonic near-field enhancement [42,43,44]. Within the investigated range, the highest enhancement was obtained for 10 PLL layers, corresponding to an effective PLL thickness of approximately 25 nm.

The enhancement factors obtained for the ZnO/PLL/Al film structures are comparable to those previously reported for Al/ZnO nanostructures with different architectures [54]. In sequentially deposited ZnO/Al films fabricated by microplotter printing, an enhancement factor of approximately 1.8 was reported [54]. However, differences in optical density, nanoparticle concentration, and the separation distance between ZnO and Al NPs may account for the different enhancement factors observed in these systems.

Importantly, the optical functionality of the PLL-coated Al nanostructures is largely preserved after aqueous exposure. As shown in Figure 6a,c, the PL enhancement factor of the ZnO/PLL(10 layers)/Al structure decreases only slightly from 2.1 to 2.0 after exposure to deionized water and remains approximately twice that of pure ZnO. The characteristic ZnO emission maximum is also preserved. Thus, PLL protects the aluminum nanostructures from degradation in water while maintaining the conditions required for plasmon-enhanced UV emission.

Finally, the Al/PLL platform was evaluated for modulation of the intrinsic fluorescence of human immunoglobulin G (IgG) (Figure 6b). In fluorescence-based bioanalysis, intrinsic emission from proteins and biological tissues can contribute to the optical background and interfere with the detection of the useful signal. Therefore, controlled quenching of protein fluorescence may provide a means to improve the signal-to-background ratio. Free IgG exhibits an emission maximum at approximately 330 nm. Immobilization of IgG on Al nanoparticles coated with 5 PLL layers decreases the fluorescence intensity by a factor of approximately 1.5, whereas 10 PLL layers result in a 2.7-fold decrease. For the 10-layer PLL coating, the emission maximum additionally shifts to approximately 322 nm. Thus, in contrast to the PL enhancement observed for ZnO, the Al/PLL platform produces pronounced fluorescence quenching of IgG. However, only PL enhancement on aluminum nanoparticles with comparable diameter has been predicted for such proteins as bovine serum albumin [68].

Overall, the obtained results demonstrate several complementary functions of PLL in plasmonic nanostructures. PLL modifies the local dielectric environment and enables spectral tuning of plasmon resonances, acts as a nanoscale spacer controlling the interaction between ZnO emitters and Al nanoparticles and thereby regulating photoluminescence enhancement, and protects reactive Al nanostructures against degradation in aqueous environments. In addition, PLL-coated Al nanostructures retain their optical functionality during aqueous processing and enable modulation of the intrinsic fluorescence of biomolecules, as demonstrated for IgG. These properties make PLL-based plasmonic structures promising for UV plasmonics, metal–semiconductor emitters, optical sensing, and fluorescence-based bioanalytical applications.

### 3.5. Discussion

Localized surface plasmon resonance (LSPR) is highly sensitive to changes in the dielectric environment within the plasmonic near field. In polymer-containing plasmonic films, spectral shifts arise mainly through two routes: changes in the effective refractive index near the metal surface and changes in plasmonic coupling caused by variations in particle/particle or particle/film separation [26,69,70,71,72,73,74,75,76,77,78,79,80,81,82]. Table 2 compares representative polymer-containing systems and the associated LSPR changes reported in the literature and obtained in this work.

The first mechanism is associated with changes in polymer thickness, swelling state, or conformation, which modify the local dielectric environment. For example, swelling of a sodium polystyrene sulfonate (PSS) film in water vapor decreases the effective refractive index by approximately 0.13–0.14 and produces a blue shift of the plasmon peak from about 610 to 600 nm [69]. In thermoresponsive poly(N-isopropylacrylamide) (PNIPAM) systems, polymer collapse upon heating expels water and increases the effective refractive index around the nanoparticles, producing a small red shift from 540 to 546 nm [70]. A similar dielectric effect is observed for Au nanoparticles embedded in poly(vinyl alcohol) (PVA): increasing the PVA thickness from approximately 30 to 330 nm progressively embeds the particles in the polymer and shifts the resonance to longer wavelengths [71]. In pH-responsive poly(2-vinylpyridine) (P2VP) brushes, swelling at low pH increases the interparticle distance and weakens plasmonic coupling, resulting in a pronounced blue shift from approximately 620–630 to 560–570 nm [72].

The second mechanism is associated with plasmonic coupling and can produce substantially larger spectral changes. In self-standing Au NP/PMMA films, the plasmon maximum shifts from approximately 530 nm in aqueous dispersion to about 680 nm in the polymer film, consistent with strong interactions in the densely packed nanoparticle layer [74]. In polymer-mediated layer-by-layer assemblies, citrate-capped Au nanospheres shift from about 520 nm in solution to approximately 680 nm after the first PEI-mediated layer and to about 715 nm after 100 layers [75]. Thermoresponsive PNIPAM-coated Au nanoparticle monolayers show the same coupling sensitivity: collapse of the polymer shell decreases the interparticle gap and shifts the plasmon band from about 640 to 690 nm [76]. Coupling between an Au nanoparticle layer and an Al film separated by a polyvinylpyrrolidone (PVP) interlayer has likewise been shown to depend strongly on the metal/film geometry [73].

Particle morphology provides an additional contribution to dielectric sensitivity. Polystyrene-grafted Au concave cubes exhibit larger LSPR shifts than flat cubes at the same polymer-shell thickness, with reported shifts of approximately 24–37 nm for concave particles compared with 14–28 nm for cubes [26]. This difference is consistent with stronger electric-field localization near sharp edges and vertices.

Compared with Au-based systems, fewer studies report directly comparable polymer-induced LSPR shifts for Ag nanoparticles. Embedding spherical Ag NPs in PMMA shifts the resonance from approximately 400 nm in water to 442 nm in the polymer matrix [82]. A related Ag/PMMA system shows a substantially larger shift, from about 387 to 480 nm, indicating that aggregation and interparticle coupling can add to the dielectric contribution [83]. The magnitude of the observed shift in our study for the Ag NPs/PLL system is significantly lower in comparison to that reported for PMMA/Ag films, although the refractive index for PMMA is identical to that of PLL in the visible range. Some other polymer-containing studies are not included in Table 2 because they do not provide a directly comparable reference-to-polymer LSPR pair [79,80], or because their primary focus is photoluminescence or other optical observables rather than an LSPR shift [84,85].

Recent plasmonic metamaterial studies further demonstrate that spectral response can be controlled not only through changes in the local dielectric environment but also through mode hybridization and nanostructure geometry. Pan et al. demonstrated coherent coupling between borophene surface-plasmon and localized surface-plasmon modes, producing multiple hybrid resonances and a tunable Rabi splitting [86]. Fu et al. showed that W–TiO2 ellipsoidal core–shell arrays support multiple localized surface plasmon-polariton and gap-plasmon resonances, whose coupling produces broadband optical absorption [87]. Although these architectures differ substantially from the PLL-coated metal nanostructures investigated here in both material composition and spectral range, they reinforce the broader principle that dielectric environment, modal coupling, and nanoscale geometry jointly determine plasmonic spectral responses.

Polymer-mediated tuning of ultraviolet plasmonic responses is less extensively documented for Pt and Al than for Au and Ag. Studies of Pt nanoparticles have mainly addressed size-dependent optical behavior of bare particles [55,84], whereas previous reports on polymer-modified Al nanostructures are limited [57].

In this context, the present work provides a controlled cross-material comparison using the same PLL deposition strategy for Al, Pt, and Ag nanostructures. Because the polymer chemistry and deposition route are kept constant, the comparison makes the material-, size-, and morphology-dependent contributions to the dielectric response more directly visible than in comparisons between unrelated literature systems.

Overall, the literature comparison indicates that LSPR shifts in polymer-containing plasmonic nanostructures are governed by three coupled factors: (1) changes in the local dielectric environment caused by polymer thickness, swelling, or conformation; (2) plasmonic coupling controlled by interparticle or particle-film separation; and (3) particle size and morphology, which determine modal structure and electric-field localization. These mechanisms can produce spectral shifts ranging from a few nanometers to well over 100 nm depending on the architecture. Against this background, the present PLL-based comparison demonstrates that the magnitude of the polymer-induced response is also strongly material dependent and cannot be attributed to refractive-index change alone.

## 4. Conclusions

A deposition strategy based on sequential Poly-L-lysine (PLL) layers was developed to control the position of plasmonic absorption peaks in nanostructures formed from different metals, including silver, platinum, and aluminum nanoparticles. The approach was demonstrated for both nanoparticle and nanowire plasmonic structures investigated in this work.

Experimental measurements and numerical calculations consistently show that increasing PLL thickness changes the local dielectric permittivity around the metal nanostructures and shifts the plasmonic absorption peak to longer wavelengths. The observed spectral shift is governed not only by the refractive index of the surrounding medium but also by the material-specific physical constants: plasma frequency and background dielectric contribution (*ε*_∞_), which is particularly critical when operating within the ultraviolet spectral range. Experimental deposition of a 25 nm PLL layer induced distinct spectral shifts depending on nanoparticle particle-size distribution and material: the red-shift magnitude of the absorption peak increased in the order Ag NPs, Pt NPs, Al NPs from 28 to 63 nm.

The Mie simulations based on silver nanoparticles show that particle size and the width of the particle-size distribution affect the spectral response. Larger particles have a greater impact on the long-wavelength part of the ensemble extinction and exhibit a more pronounced red shift, while the increasing contribution of retardation and multipolar effects leads to the appearance of higher-order plasmonic modes.

The PLL deposition approach can also be used to control the separation distance between an emitter and a plasmonic nanoparticle, as demonstrated for the UV photoluminescence of ZnO at 377 nm. Experimentally, the PL enhancement factor increased from 1.4 for ZnO/Al without PLL to 1.9 for 5 PLL layers and 2.1 for 10 PLL layers. This behavior is supported by electromagnetic modeling of the local field around an Al nanoparticle covered by a native aluminum oxide shell and a PLL layer of variable thickness. The calculations indicate local-field enhancement of up to approximately 2.8 with PLL thicknesses of 2 and 40 nm.

This study demonstrates an approach enabling the preservation of aluminum nanoparticles’ optical properties in an aqueous environment for up to 340 min. The polymer suppresses oxidation and aluminum hydroxide formation, providing stability of the absorption-peak position and the ability of Al nanostructures to enhance the photoluminescence of ZnO nanocrystals. These findings establish PLL-coated Al nanostructures’ potential utility for bio-imaging and biosensor applications, since proteins and amino acids are predominantly encountered in aqueous environments. We demonstrated PL quenching of IgG emission at 330 nm based on the obtained Al nanostructures, which is relevant for reducing parasitic background signals in fluorescence-based bioimaging.

Overall, PLL acts as a controllable dielectric and protective interlayer that combines three functions within a single platform: spectral tuning of plasmonic resonances, control of emitter–metal separation distance, and stabilization of Al nanostructures in aqueous environments. This combination makes the developed approach promising for UV plasmonics, optical sensing, metal–semiconductor emitters, and fluorescence-based bioanalytical systems.

## Figures and Tables

**Figure 1:**
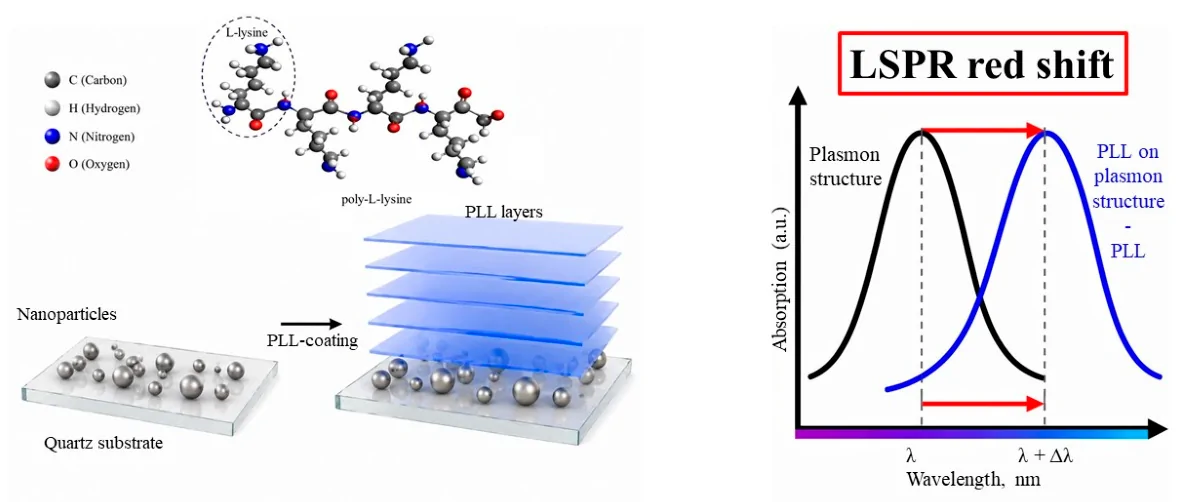
Schematic illustration of PLL-induced LSPR red shift. The red arrow indicates the shift of the LSPR peak toward longer wavelengths following PLL coating.

**Figure 2:**
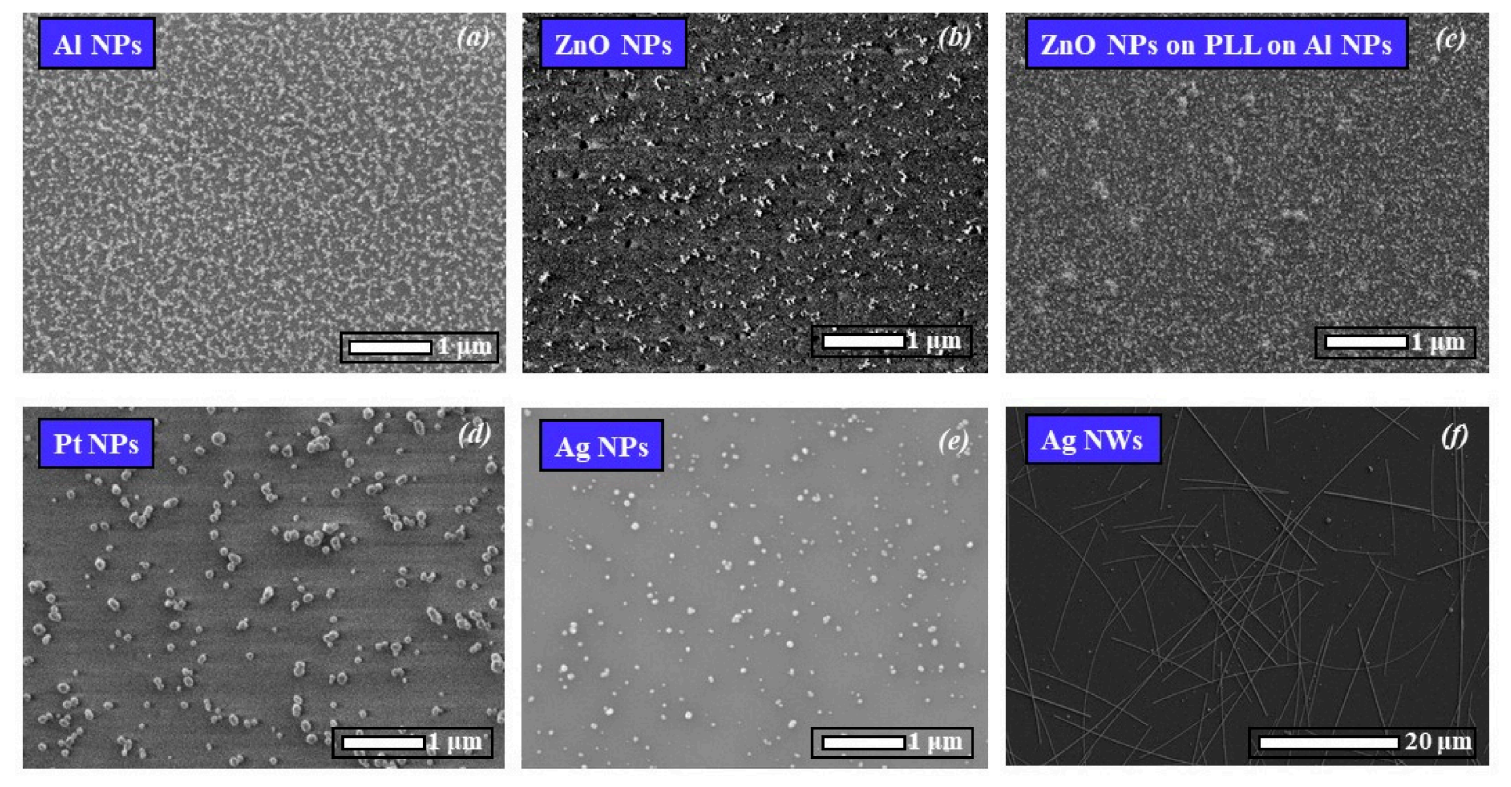
SEM images of bare Al NPs (**a**), ZnO NPs (**b**), and the three-layer system ZnO/PLL/Al, where ZnO NPs are deposited on PLL-coated Al NPs (**c**), Pt NPs (**d**), Ag NPs (**e**), and Ag NWs (**f**).

**Figure 3:**
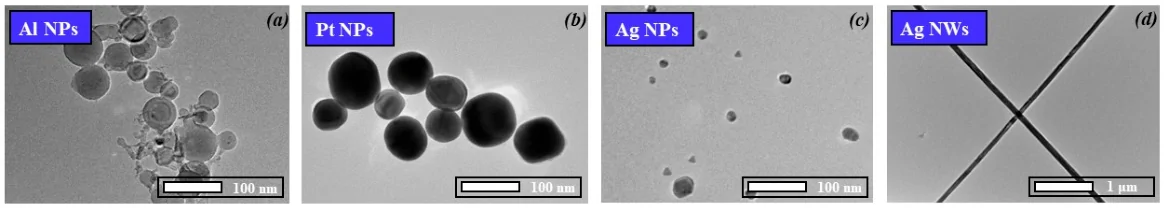
TEM images of Al NPs (**a**), Pt NPs (**b**), Ag NPs (**c**), and Ag NWs (**d**).

**Figure 4:**
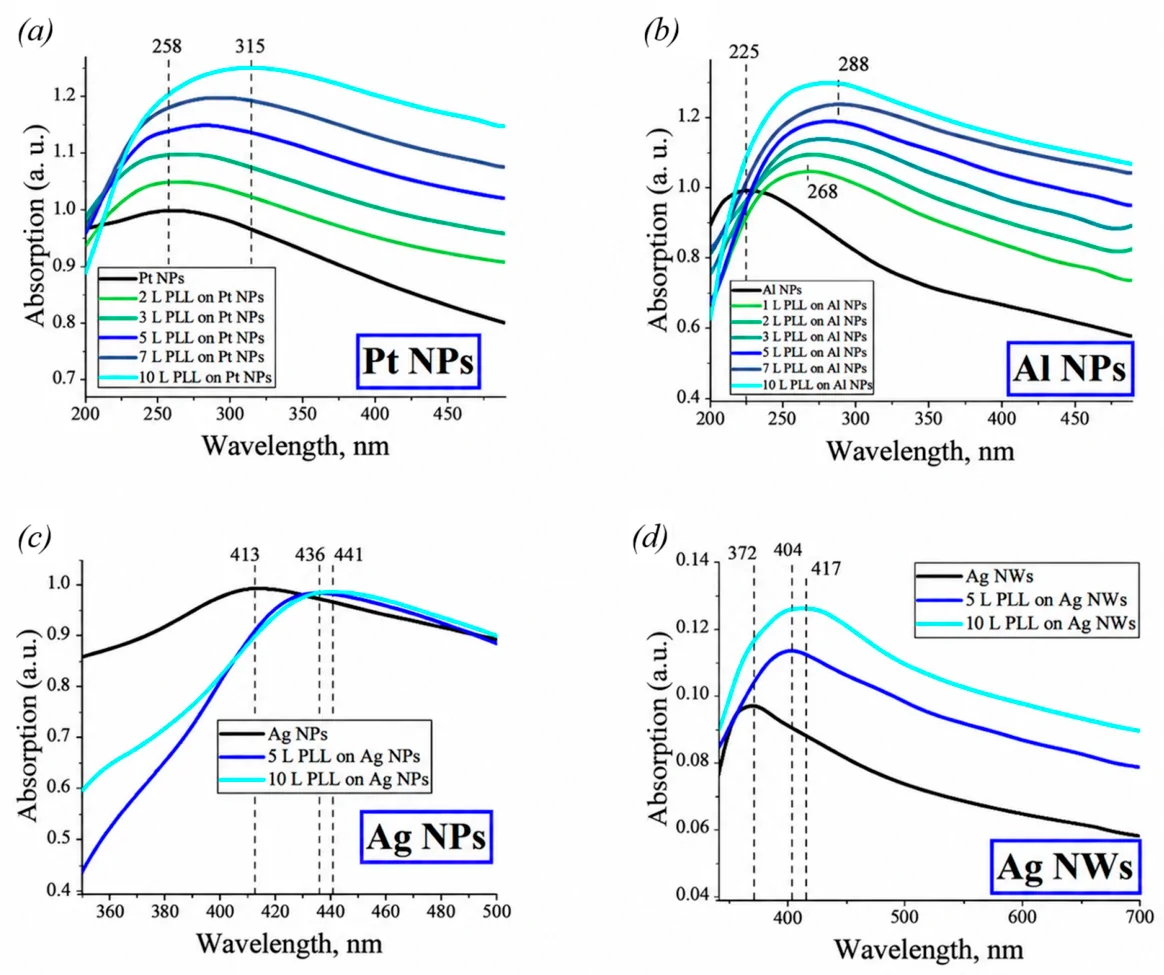
PLL-induced red shift of localized surface plasmon resonance in plasmonic nanostructures. UV–vis absorption spectra of (**a**) Pt NPs, (**b**) Al NPs, (**c**) Ag NPs, and (**d**) Ag NWs before and after deposition of PLL layers.

**Figure 5:**
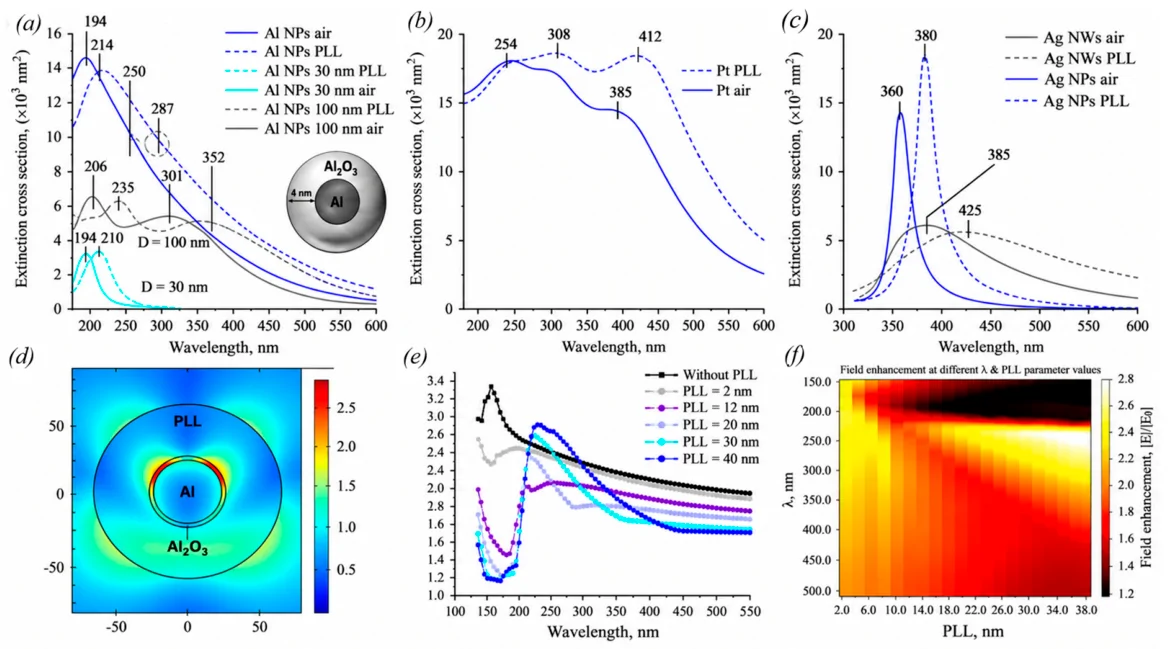
Numerical simulation of extinction spectra and local electric-field enhancement in plasmonic nanostructures: extinction cross section of (**a**) Al NPs, (**b**) Pt NPs, and (**c**) Ag NPs and Ag NWs of different sizes in air and PLL; (**d**) spatial distribution of the electric-field enhancement around an Al/Al_2_O_3_ nanoparticle coated with PLL; (**e**) wavelength-dependent electric-field enhancement for different PLL thicknesses; (**f**) electric-field enhancement as a function of extinction wavelength and PLL thickness.

**Figure 6:**
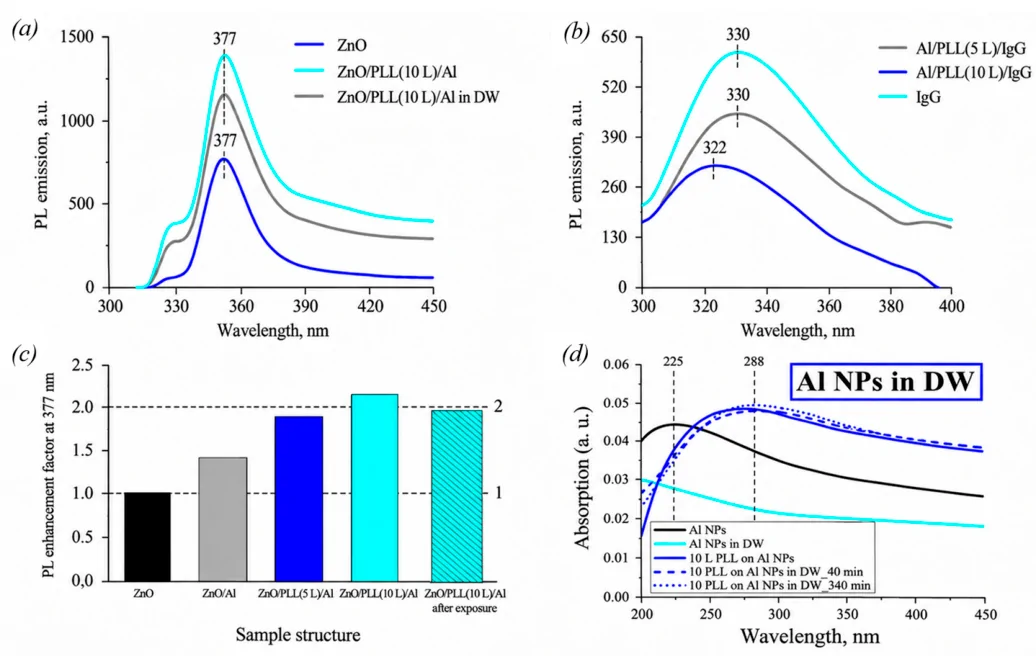
Optical response and aqueous stability of PLL-functionalized Al-based nanostructures. (**a**) PL spectra of ZnO and ZnO/PLL(10 layers)/Al before and after exposure to deionized water (DW); (**b**) PL spectra of free IgG and IgG immobilized on Al nanoparticle films coated with 5 and 10 PLL layers; (**c**) PL enhancement factors at 377 nm for ZnO, ZnO/Al, ZnO/PLL(5 layers)/Al, and ZnO/PLL(10 layers)/Al, including ZnO/PLL(10 layers)/Al after exposure to DW; (**d**) UV–vis absorption spectra of bare Al NPs and PLL-coated Al NPs before and after immersion in DW (40 and 340 min).

**Table 1:** Comparison of material parameters and resonance shifts for the studied plasmonic nanostructures.

Parameter	Al	Pt	Ag NPs	Ag NWs
Plasma energy, *E_p_* (eV) [59]	14.98	9.59	9.01	
Proportionality coefficient according to Equation (8), ε∞ = 1	0.025	0.040	0.042	
Mie-simulation shift, nm	20 37	8 10 27	20	40
Mie-simulation peak position, nm (air → PLL)	194 → 214 ~250 → 287	254 → 262 298 → 308 385 → 412	360 → 380	385 → 425
Experimental shift, nm (10 PLL layers)	63	57	28	45
Experimental peak position, nm (air → PLL)	225 → 288	258 → 315	413 → 441	372 → 417
Mean diameter, nm	55 ± 25	105 ± 33	36 ± 13	84 ± 21

**Table 2:** Comparison of LSPR shifts in polymer-coated plasmonic nanostructures reported in the literature and obtained in this work.

Ref.	Material	NP Size	Polymer	Thickness	Reference LSPR Peak (nm)	Shifted LSPR Peak/Condition (nm)
[69]	Au NPs island film	5 nm	PS; PSS	55 nm; 85 nm	545	585–595; ~575 after chloroform-vapor swelling (blue shift)
[70]	Au NPs	30 nm	PNIPAM brush	40–130 nm	—	540 (20 °C); 546 (40 °C)
[71]	Au NP	diameter ~80 nm (radius 40 ± 8 nm)	PVA	~30 or ~330 nm	—	560–600 (~30 nm film); ~662 (~330 nm film)
[72]	Au NPs (colloidal) + island films	10.7 nm	P2VP brush	7.4–63 nm	518	620–630 (pH 5.5); 560–570 (pH 2.5)
[73]	Au NPs coupled to Al film	14 nm (PVP-stabilized); 91 nm (bare)	PVP interlayer	Not reported	525–530	550–580; 580–590 for 62 nm Al film
[74]	Au NPs	~20 nm	PMMA film	~270 nm	~530 (aqueous dispersion)	~680 (Au NP/PMMA film)
[75]	Au spheres; rods; prisms; octahedra	20 nm; 100 × 15 nm; 90 nm; 50 nm	PEI, PVP, PSS, pDADMAC; layer-by-layer films	—	Spheres: ~520 (solution)	Spheres/PEI: ~680 (1 layer), ~715 (100 layers); rods/PSS: >900
[76]	Au NPs	~20 nm	PNIPAM-grafted shell	—	—	~640 (25 °C); ~690 (35 °C)
[26]	Au cubes and concave cubes	50–90 nm	Polystyrene (PS) shell	~20 nm	Cubes: 539–587; concave: 607–669	Cubes: 554–615; concave: 631–706
[77]	Au@P4VP NPs	~5 nm	P4VP + PS-b-P4VP	Several tens of nm	527	525 after spin-coating; 531 after HCl treatment
[78]	Au core	4.2 nm	PS + PNIPAM	Not reported	514	568; 560 upon compression
[81]	Au NPs	16 nm	PVP; PDMS	70 nm; 1 mm	—	PVP: 535–542; PDMS: 542–537
[82]	Ag NPs, spherical	5.6 nm average	PMMA film	~685 µm	~400 (water)	442 (PMMA film)
[83]	Ag NPs, spherical	5.6 nm average	PMMA film	~25 µm	~387 (water)	~480 (PMMA film; Figure 1)
This work	Al NPs, spherical	55 ± 25 nm	PLL	25 nm	225	288 (25-nm PLL)
This work	Pt NPs, quasi-spherical	105 ± 33 nm	PLL	25 nm	258	315 (25-nm PLL)
This work	Ag NPs, spherical	36 ± 13 nm	PLL	25 nm	413	441 (25-nm PLL)
This work	Ag NWs	Diameter 84 ± 21 nm; length ~15 µm	PLL	25 nm	372	417 (25-nm PLL)

## Data Availability

The raw data supporting the conclusions of this article will be made available by the authors on request.

## Supplementary Materials

The following supporting information can be downloaded at: https://www.mdpi.com/article/10.3390/nano16211416/s1, Figure S1: UV–vis absorption spectra of PLL films consisting of 1, 5, and 10 sequentially deposited layers; Figure S2: Particle-size distributions obtained from TEM images: (a) Pt nanoparticles, (b) Al nanoparticles, (c) Ag nanoparticles, (d) Ag nanowire diameters, and (e) Ag nanowire lengths; Figure S3: Refractive index of Poly-L-lysine as a function of photon energy, calculated using the Cauchy model; Figure S4: Dependence of the localized surface plasmon resonance (LSPR) wavelength shift of Al nanoparticles on the thickness of sequentially deposited Poly-L-lysine (PLL) layers. Black squares represent experimental data, while the blue line represents the exponential saturation fit; Figure S5: Dependence of the localized surface plasmon resonance (LSPR) wavelength shift of Pt nanoparticles on the thickness of sequentially deposited Poly-L-lysine (PLL) layers. Black squares represent experimental data; Table S1: Thickness of Poly-L-lysine (PLL) films as a function of the number of sequentially spin-coated layers, determined by spectroscopic ellipsometry.
