# Supplementary material for: Multifunctional Role of Poly-L-Lysine in Plasmonic Metal Nanostructures
Source: Nanomaterials (Basel). 2026 Nov 4;16(21):1416. doi: 10.3390/nano16211416 (PMC13765144; doi:10.3390/nano16211416)
Supplement: Supplementary file 1 [file nanomaterials-16-01416-s001.zip › nanomaterials-4595215-supplementary.pdf]

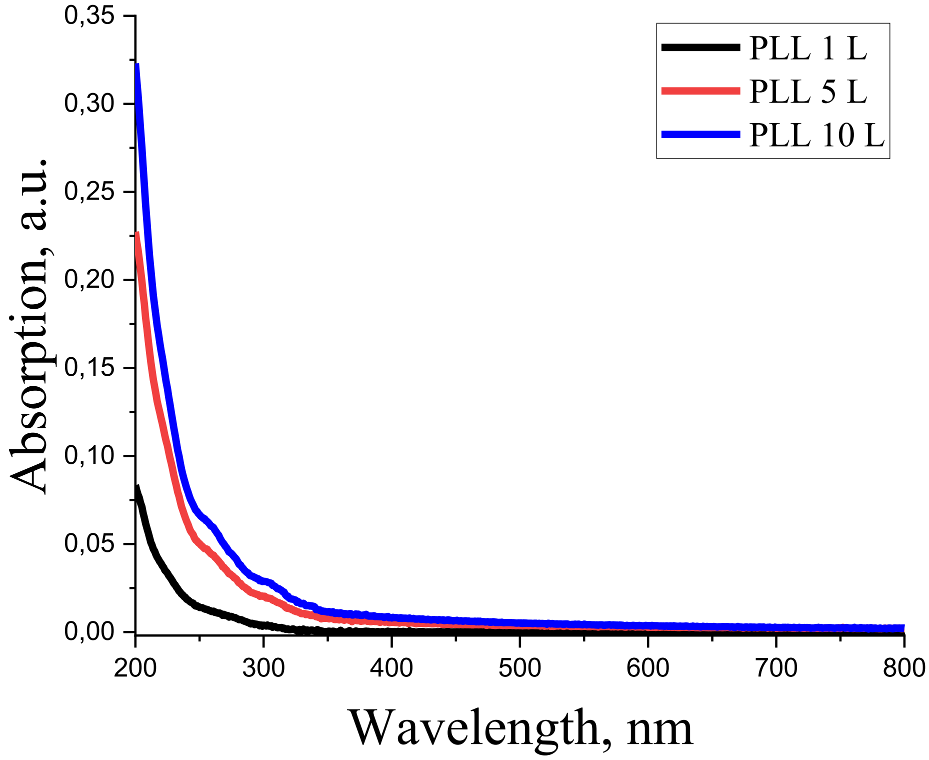

Figure S1. UV-vis absorption spectra of PLL films consisting of 1, 5, and 10 sequentially deposited layers.

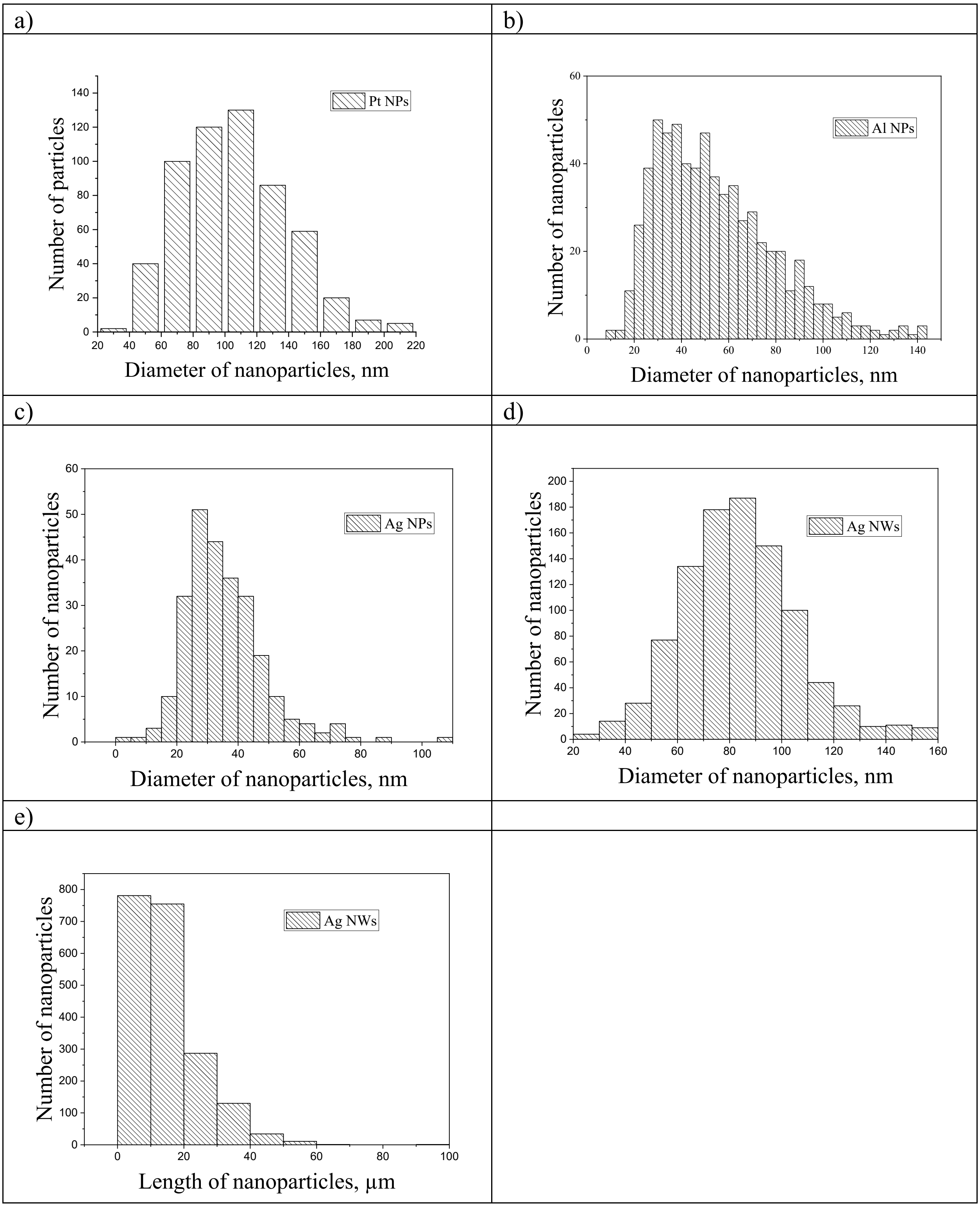

Figure S2. Particle-size distributions obtained from TEM images: (a) Pt nanoparticles, (b) Al nanoparticles, (c) Ag nanoparticles, (d) Ag nanowire diameters, and (e) Ag nanowire lengths.

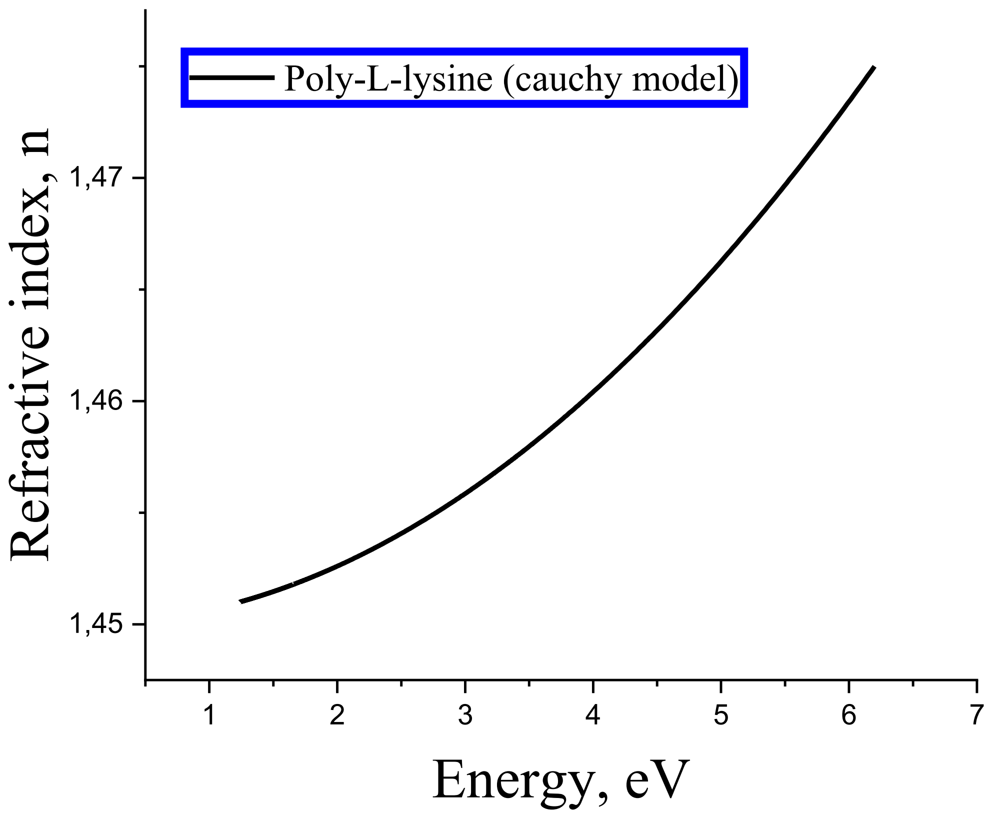

Figure S3. Refractive index of Poly-L-lysine as a function of photon energy, calculated using the Cauchy model.

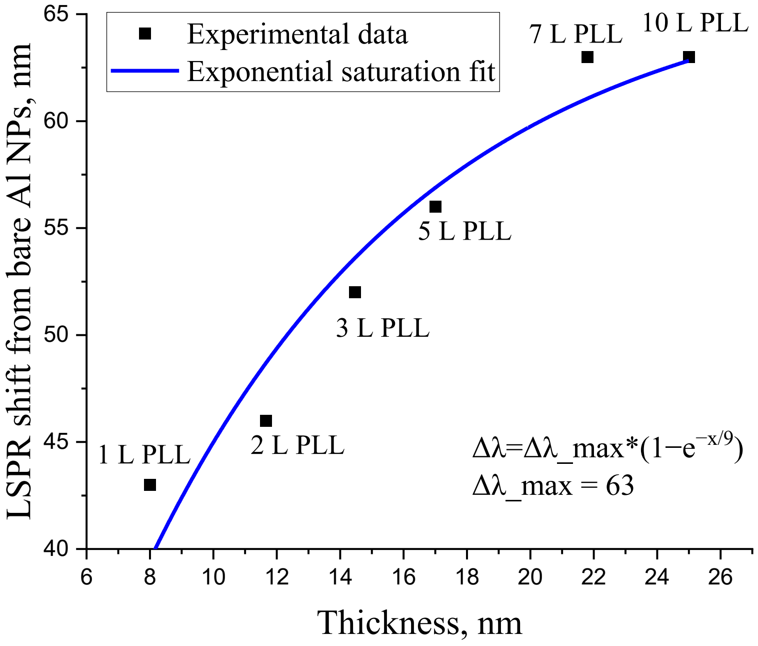

Figure S4. Dependence of the localized surface plasmon resonance (LSPR) wavelength shift of Al nanoparticles on the thickness of sequentially deposited Poly-L-lysine (PLL) layers. Black squares represent experimental data, while the blue line represents the exponential saturation fit.

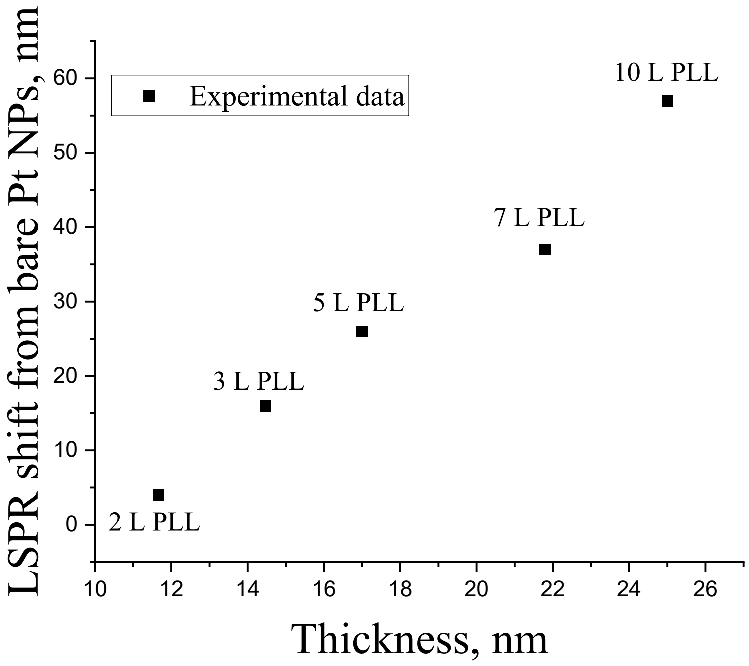

Figure S5. Dependence of the localized surface plasmon resonance (LSPR) wavelength shift of Pt nanoparticles on the thickness of sequentially deposited Poly-L-lysine (PLL) layers. Black squares represent experimental data.

Table S1. Thickness of Poly-L-lysine (PLL) films as a function of the number of sequentially spin-coated layers, determined by spectroscopic ellipsometry.

Number of PLL layers	Thickness (nm)
1	8
2	11.66
3	14.47
5	17
7	21.8
10	25

Mean Particle Dimensions

The mean dimensions determined from the TEM images were 105 ± 33 nm for Pt nanoparticles, 55 ± 25 nm for Al nanoparticles, and 36 ± 13 nm for Ag nanoparticles. Ag nanowires exhibited an average diameter of 84 nm and an average length of approximately 15 µm.
